# MiR-150 in HTLV-1 infection and T-cell transformation

**DOI:** 10.3389/fimmu.2022.974088

**Published:** 2022-08-16

**Authors:** Donna M. D’Agostino, Vittoria Raimondi, Micol Silic-Benussi, Vincenzo Ciminale

**Affiliations:** ^1^ Department of Biomedical Sciences, University of Padova, Padova, Italy; ^2^ Istituto Oncologico Veneto (IOV)- Istituto di Ricovero e Cura a Carattere Scientifico (IRCCS), Padova, Italy; ^3^ Department of Surgery, Oncology and Gastroenterology, University of Padova, Padova, Italy

**Keywords:** HTLV-1, miR-150, T-cells, microRNA, adult T-cell leukemia/lymphoma, leukemia, MYB

## Abstract

Human T-cell leukemia virus-1 (HTLV-1) is a retrovirus that persistently infects CD4+ T-cells, and is the causative agent of adult T-cell leukemia/lymphoma (ATLL), tropical spastic paraparesis/HTLV-1-associated myelopathy (TSP/HAM) and several inflammatory diseases. T-cell transformation by HTLV-1 is driven by multiple interactions between viral regulatory proteins and host cell pathways that govern cell proliferation and survival. Studies performed over the last decade have revealed alterations in the expression of many microRNAs in HTLV-1-infected cells and ATLL cells, and have identified several microRNA targets with roles in the viral life cycle and host cell turnover. This review centers on miR-150-5p, a microRNA whose expression is temporally regulated during lymphocyte development and altered in several hematological malignancies. The levels of miR-150-5p are reduced in many HTLV-1-transformed- and ATLL-derived cell lines. Experiments in these cell lines showed that downregulation of miR-150-5p results in activation of the transcription factor STAT1, which is a direct target of the miRNA. However, data on miR-150-5p levels in freshly isolated ATLL samples are suggestive of its upregulation compared to controls. These apparently puzzling findings highlight the need for more in-depth studies of the role of miR-150-5p in HTLV-1 infection and pathogenesis based on knowledge of miR-150-5p-target mRNA interactions and mechanisms regulating its function in normal leukocytes and hematologic neoplasms.

## Introduction

Human T-cell leukemia virus-1 (HTLV-1), the first retrovirus identified as pathogenic in humans (1), infects 5-10 million persons worldwide (2). HTLV-1 is the causative agent of adult T-cell leukemia/lymphoma (ATLL), tropical spastic paraparesis/HTLV-1-associated myelopathy (TSP/HAM) and several inflammatory diseases (3). The unique characteristics of HTLV-1 replication and persistence have thus far impeded the development of effective strategies to control infection and treat HTLV-1-associated diseases [reviewed by (4, 5)].

HTLV-1 codes for the regulatory/accessory proteins Tax, Rex, p30, p13, p12, and HBZ in addition to the Gag, Env and Pol genes common to all retroviruses [reviewed by (6, 7)]. Tax and Rex are essential for productive viral infection, with Tax driving transcription of the plus-strand genome [reviewed by (8)] and Rex functioning as an RNA escort that facilitates exit of plus-strand viral transcripts from the nucleus [reviewed by (9)].

CD4+ CD25+ T-cells are the main targets of HTLV-1 infection and transformation *in vivo* (10). In the current model of ATLL pathogenesis, Tax is considered to be the primary driver of neoplastic transformation while HBZ is essential for maintaining the transformed cell population [reviewed by (11, 12)].

## HTLV-1 and the cellular miRNA network in infected T-cells and ATLL cells

microRNAs (miRNAs) are small RNAs of about 22 nt that induce degradation and/or block translation of target mRNAs after binding to complementary sequences in the mRNA’s 3’UTR. miRNA-mRNA interactions thus contribute to the precise control of gene expression in physiological and pathological settings including cancer [reviewed by (13)].

The role of miRNAs in HTLV-1 infection and disease has been investigated using PBMC from ATLL patients and asymptomatic carriers, cell lines and clones stabilized from ATLL cells or infected PBMC, and HTLV-1-transformed cell lines generated by cocultivating normal cells with patients’ infected cells. Control cells included normal PBMC, isolated CD4+ T-cells and the T-cell acute lymphoblastic leukemia (T-ALL)-derived cell line Jurkat. Results of these studies yielded many up-and downregulated miRNAs [reviewed by (14)].

Both Tax and HBZ directly influence the expression of specific miRNAs [reviewed by (15)]. Tax and HBZ also have general disruptive effects on miRNA expression through Tax-mediated ubiquitination and degradation of Drosha (16) and HBZ-mediated repression of Dicer expression (17). Dicer activity is also reduced through interactions with Rex (18).

Only a few miRNA-mRNA interactions have been described in the context of HTLV-1-transformed cells. miR-93 and miR-130b, identified as upregulated in ATLL samples and ATLL-derived cell lines, target the mRNA coding for the pro-apoptotic protein TP53INP1 (19). Two miRNAs upregulated by HBZ, miR-17 and miR-21, repress expression of the DNA-binding protein OBFC2A (gene symbol NABP1) (20). miR-34a-5p is overexpressed in HTLV-1-transformed cell lines and ATLL samples (19, 21); its targets identified in HTLV-1-transformed cell lines include the protein deacetylase SIRT1, the pro-apoptotic protein BAX, and the anti-apoptotic protein BIRC5 (Survivin) (21). miRNA profiling of a large panel of ATLL samples and normal CD4+ T-cell controls revealed downregulation of many miRNAs in ATLL cells (22). The most dramatically downregulated miRNA, miR-31, is silenced by genetic deletions and Polycomb-mediated repression. Downregulation of miR-31 increases levels of its target MAP3K14 (NIK), a positive regulator of the noncanonical NF-ĸB pathway (22). Bellon et al. showed that miR-124a is downregulated in ATLL samples, ATLL-derived cell lines and chronically infected cell lines compared to normal PBMC due to promoter methylation, and identified STAT3 as a miR-124a target (23).

miR-150-5p, one of the most highly expressed miRNAs in normal naïve CD4+ T-cells (see below), stands out among the many miRNAs that have been identified as deregulated by HTLV-1, as it was initially reported to be downregulated in HTLV-1-transformed cell lines and ATLL-derived cell lines but upregulated in ATLL samples compared to normal PBMC (19) and CD4+ T-cells (24). On the other hand, the 2012 study by Yamagishi et al. (22) did not identify miR-150 as up- or downregulated in ATLL samples vs. CD4+ controls using a stringent > 5-fold cutoff for differential expression.

RNAseq analyses confirmed significant downregulation of miR-150-5p in the HTLV-1-transformed cell lines C91PL and MT-2 compared to normal CD4+ T-cells (25). miRBase (version 22.1; https://www.mirbase.org) indicates a strong bias for expression of miR-150-5p compared to miR-150-3p; the text below refers to miR-150-5p as miR-150.

In an analysis of miR-150 and miR-223 in additional HTLV-1-transformed cell lines and ATLL cell lines (IL-2-independent or IL-2-dependent) compared to Jurkat cells, Moles et al. (26) detected reduced levels of both miRNAs in the transformed cell lines and the IL-2-independent ATLL cell lines, while the IL-2-dependent ATLL cell lines showed reduced levels of miR-223, but increased levels of miR-150. Analyses of the cell lines cultured with or without IL-2 indicated a link between miR-150 expression and IL-2 stimulation/dependence. Functional assays showed that miR-150 and miR-223 directly target STAT1. Ectopic expression of miR-150 or miR-223 interfered with proliferation of an HTLV-1-transformed cell line, indicating a tumor suppressor function (26).

Recent RNAseq analyses of PBMC from asymptomatic HTLV-1-infected patients (ASP) and control PBMC identified many differentially expressed small RNAs, including upregulated miR-150, in the ASP samples (27). Additional studies that included PBMC from ATLL patients detected higher levels of miR-150 in ATLL vs. both ASP and control PBMC (28). These findings would be strengthened by comparison of miRNAs in purified CD4+ cells, since PBMC preparations from ATLL samples contain a preponderance of infected CD4+ neoplastic cells compared to ASP or control PBMC samples.

Given the importance of miR-150 in normal lymphocyte development and its altered expression in a variety of neoplasms (29, 30), the expression of miR-150 and its mRNA targets in the context of HTLV-1 infection and disease merit further investigation. The following sections provide information on the role of miR-150 in normal CD4+ T-cells and selected hematologic malignancies that could help guide the design of further investigations of this miRNA as a potential actor in HTLV-1 replication and pathogenesis.

### miR-150 and MYB

miR-150 levels are temporally regulated during B- and T-cell maturation. The first detailed miRNA profiling analyses of murine lymphocyte populations revealed abundant levels of miR-150 in mature B-cells and CD4+ T-cells compared to pro-B cells and thymocytes (31). *In vitro* activation of mature naïve CD4+ T-cells and CD8+ T-cells is accompanied by substantial alterations in the pattern of miRNA expression, including a rapid decline in miR-150 levels (31–33).

HTLV-1-infected cells and ATLL cells frequently express markers of regulatory T-cells (Tregs) including GITR (34), CD45RO (35) and FOXP3 (36). Early studies of murine lymphocytes showed that miR-150 is much less abundant in Tregs compared to helper CD4+ T-cells, and that forced expression of FOXP3 In CD4+ T-cells leads to downregulation of miR-150 (32).

Forced premature expression of miR-150 in hematopoietic stem/progenitor cells blocks B-cell development at the pro-B stage without overt disruption of T- and myeloid populations (37), while miR-150-knockout mice develop an expanded population of the B-cell subtype B1 without substantial effects on other B-cell subtypes or T-lymphocytes (38). The studies of murine B-cell maturation led to the identification of the transcription factor MYB (c-Myb) as a target of miR-150 (38). MYB plays a key role in the regulation of hematopoietic cell development and turnover, and it is deregulated in cancer cells [reviewed by (39)].

Recent miR-150-RNA-pull-down experiments in resting and *in vitro*-activated human CD4+ T-cells yielded the MYB transcript and many additional bound mRNAs, including PIK3R1 (phosphoinositide-3-kinase regulatory subunit 1), HNRNPAB (heterogeneous nuclear ribonucleoprotein AB), and PDAP1 (PDGFA-associated protein 1) (40). Results of CRISPR/Cas-9 editing experiments verified the relevance of miR-150, MYB and PDAP1 in regulating the activation state of CD4+ T-cells (40).

Although the potential for miR-150 to regulate MYB in the context of HTLV-1 has not been investigated, MYB function and expression are known to be altered in HTLV-1-infected cells through the actions of Tax and HBZ, with contrasting effects: while Tax represses expression of MYB (41, 42) and competes with MYB for binding to the transcription coactivators CBP/p300 (43), HBZ displaces Tax from p300/CBP and promotes MYB-CBP/p300 binding (44).

**Figure 1:**
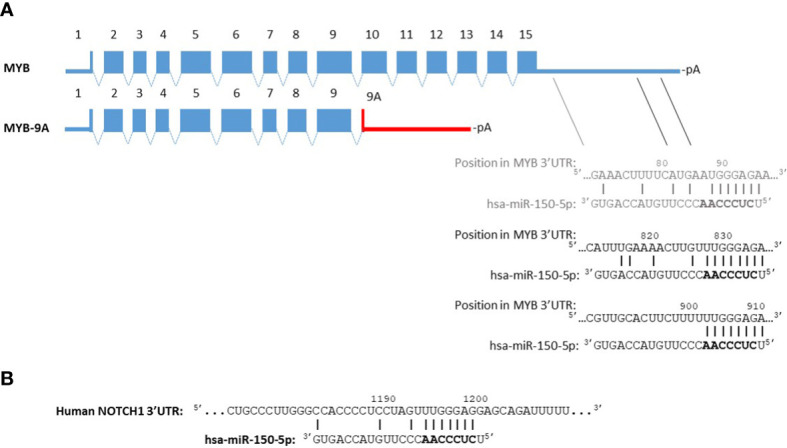
miR-150 binding site predictions for MYB-9A and NOTCH1. Panel **(A)** The alternatively spliced transcript coding for full-length MYB (640 amino acids) contains 15 exons. TargetScan (46) predicts 3 binding sites for miR-150 in the MYB 3’UTR (ENST00000367814.4, 1193 nt). The second and third sites were found to be functionally relevant by Xiao et al. (38). The 3’UTR of MYB-9A (402 amino acids) has not been experimentally verified. The red line indicates a predicted 3’UTR based on the presence of a consensus sequence for polyadenylation (47) downstream of the MYB-9A stop codon. The miRNA target prediction tools STarMir (48) and RNA22 (49) do not identify miR-150-5p binding sites in this sequence. Panel **(B)** An NCBI BLAST search of the region spanning the functionally relevant miR-150 binding site in the murine NOTCH1 3’UTR characterized by Deng et al. (50) identified a highly similar sequence in the human NOTCH1 3’UTR. This segment contains a potential binding site for miR-150-5p according to TargetScan analysis of the NOTCH1 3’UTR (ENST00000277541.6, 1627 nt). TargetScan Release 8.0: https://www.targetscan.org/vert_80/, STarMir: http://sfold.wadsworth.org/, RNA22 version 2.0: http://cm.jefferson.edu/rna22/Interactive/.

MYB is upregulated in ATLL samples compared to CD4+ T-cell controls (45). ATLL samples exhibit very high expression of MYB and in particular the splicing variant MYB-9A, a shorter protein isoform that is more active than full-length MYB. Both MYB and MYB-9A were shown to activate the NF-κB pathway, and knockdown of total MYB or MYB-9A alone induces apoptosis of ATLL cells (45). As depicted in **Figure 1A**, the predicted 3’UTR region of the MYB-9A mRNA lacks 2 functionally verified binding sites for miR-150 that are present in the full-length MYB transcript (38), suggesting that the MYB-9A mRNA in ATLL cells might bypass negative regulation by miR-150.

### miR-150 and NOTCH

An analysis of miRNA expression during human thymocyte maturation identified miR-150 among the most strongly upregulated miRNAs in the transition from CD4+CD8+ double-positive (DP) cells to mature CD4+ or CD8+ single-positive (SP) cells, and confirmed its sustained expression in mature circulating CD4+ and CD8+ SP populations (51). Integration of gene expression data obtained for thymocyte populations with miRNA target predictions led to the identification of NOTCH3 as a miR-150 target. Forced expression of miR-150 in T-ALL cell lines reduced the levels of NOTCH3, slowed cell proliferation and induced apoptotic death (51).

The NOTCH family proteins (NOTCH1-4) are cell-surface, transmembrane proteins that, after interacting with a NOTCH ligand (DLL1, DLL3, DLL4, JAG1, JAG2), undergo proteolytic cleavage of their cytoplasmic tails. The resulting Notch intracellular domain (NICD) peptide transfers to the nucleus, where it regulates transcription of target genes. NOTCH signaling regulates development and cell fate, including that of T- and B-cells, and is frequently deregulated in hematologic- and solid cancers [reviewed by (52)].

The NOTCH pathway is activated through diverse mechanisms in ATLL cells, including oncogenic mutations in NOTCH1 (53, 54), overexpression of JAG1 (55), a Tax-mediated increase in the half-life of the NICD (56), inactivating mutations of FBXW7, a ubiquitin ligase that directs NICD degradation (57), and mutations in NOTCH pathway-regulating genes ATXN1 and ZFP36L2 (54).

A recent study of pathways involved in the activation of murine macrophages demonstrated that miR-150 directly targets NOTCH1 (50). As shown in **Figure 1B**, the human NOTCH1 3’UTR contains a predicted binding site for miR-150 that is highly similar to the murine sequence. The possible impact of miR-150 on NOTCH signaling in HTLV-1-infected cells through its repression of NOTCH1 and/or NOTCH3 thus merits investigation.

### miR-150 and GLUT1

The extent of miR-150 downregulation upon stimulation of naïve T-cells and downstream effects depend on the activating stimulus. Analyses of the response of CD4+ T-cells to stimulation with either anti-CD3/CD28 or anti-CD3/CD46 antibodies revealed a more pronounced downregulation of miR-150 in CD3/CD46 stimulated cells, which was paralleled by a more substantial increase in MYB levels (58). Further characterization of the activated cells identified the glucose transporter SLC2A1 (GLUT1) as a miR-150 target (58). These findings are of interest in the context of HTLV-1 infection, as GLUT1 plays a key role in the binding/entry phase of HTLV-1 infection (59). The observation that GLUT1 is needed to set up a metabolic profile that supports HIV-1 replication in CD4+ T-cells (60) suggests that regulation of the miR-150/GLUT1 balance might influence HTLV-1 replication at both the entry and post-entry phases.

### miR-150 and AKT/mTOR signaling

miR-150 regulates the maturation and activity of NK cells (61, 62), and is frequently downregulated in NK/T lymphomas (63, 64). Forced expression of miR-150 in NK/T lymphoma cells results in reduced cell proliferation, increased apoptosis and a senescent phenotype. These effects were attributed in part to miR-150-mediated targeting of DKC1 (dyskerin pseudouridine synthase 1) and AKT2 (AKT serine/threonine kinase 2), both of which regulate telomerase activity (63). A subsequent study identified both AKT2 and AKT3 as miR-150 targets and showed that reintroduction of the miRNA into NK/T lymphoma cells increases their sensitivity to killing by ionizing radiation (64).

AKT is activated in ATLL cells and HTLV-1-transformed cell lines (65). Activated AKT feeds into the mTOR signaling network, which plays a central role in nutrient sensing, metabolism, and cell growth, and is frequently deregulated in cancer (66). Studies of T-ALL cells indicated a role for the AKT/mTOR pathway in downregulating miR-150 (67). Treatment of the T-ALL cell line Jurkat with the mTORC1-inhibitor rapamycin resulted in an increase in the levels of miR-150 and a block in the cell cycle; forced expression of miR-150 plus rapamycin augmented the anti-proliferative effect (67). Experiments performed in the myeloid leukemia cell line K562 led to the identification of FOXO4, TET3, PRKCA and EIF4B as direct miR-150 targets (68). The fact that EIF4B is an important downstream participant in mTOR signaling suggests that mTOR and miR-150 functionally antagonize each other. These observations are of interest, as both Tax and HBZ stimulate the mTOR pathway (69, 70). HTLV-1-transformed cell lines and ATLL samples respond to rapamycin and its analogue everolimus with growth arrest (65, 71), with long-term everolimus treatment inducing a senescent phenotype followed by apoptosis (71). It would thus be interesting to evaluate the effects of mTOR inhibition on miR-150 expression in ATLL/HTLV-1-infected cells and the impact of combining mTOR inhibitors with a miR-150 mimic on cell senescence/death.

Integration of gene expression data from rapamycin-treated/control Jurkat cells and miRNA target predictions suggested a role for miR-150 in cell cycle regulation, and led to the identification of CDK2 (cyclin-dependent kinase 2) as a direct miR-150 target (67). It is noteworthy that CDK2 was found to be significantly upregulated in a panel of ATLL-derived and HTLV-1-transformed cell lines compared to activated T-cell controls (72); low miR-150 levels might thus contribute to sustain growth of HTLV-1-infected cells by relaxing control of CDK2 expression.

### miR-150 and CCR6

Cutaneous T-cell lymphoma (CTCL) defines a variety of neoplasms characterized by the accumulation of mature T-cells in the skin, and includes ATLL cases with cutaneous homing [reviewed by (73)]. An analysis of a panel of CTCL cell lines and primary CTCL samples (including ATLL lymph node biopsies) revealed significant downregulation of miR-150 in CTCL cells compared to normal CD4+ T-cells (74). Ectopic expression of miR-150 in CTCL cells reduced their growth in immunodeficient mice and interfered with their migration/dissemination properties. These effects were linked to miR-150-mediated downregulation of CCR6, a chemokine receptor recognized by CCL20 (74). Treatment with histone deacetylase inhibitors resulted in a decline in CCR6 levels accompanied by changes in expression of many miRNAs, including upregulation of miR-150 (75). The survival of CTCL-inoculated mice was prolonged by systemic treatment with a miR-150 mimic or a siRNA against CCR6, suggesting a possible therapeutic application for miR-150 (75). These findings are of interest, as an earlier study of inflammatory signals associated with HTLV-1 pathogenesis had shown that HTLV-1-transformed cell lines express high levels of both CCR6 and its ligand, CCL20, which is upregulated by Tax through the NF-κB pathway (76). Consistent with these observations, expression of CCR6 was found to be elevated in PBMC from TSP/HAM patients and asymptomatic carriers compared to healthy controls (77). Taken together, these observations suggest that low miR-150 levels may contribute to sustain CCR6 expression in HTLV-1-infected cells.

### miR-150 and viral transcripts

Cellular miRNAs can potentially influence the retroviral life cycle by directly targeting viral transcripts. miR-150, together with miR-28, miR-125b, miR-223, and miR-382, contribute to maintain HIV-1 latency in resting CD4+ T cells by binding to the viral 3’ UTR, resulting in repression of viral gene expression (78). A search for interactions between miRNAs and HTLV-1 transcript showed that miR-28-3p binds to the unspliced viral RNA (genomic and coding for Gag-Pro-Pol) and singly spliced Env mRNA (79). This interaction blocks viral replication at the reverse transcription step (79). The HTLV-1 plus- and minus strand primary transcripts contain many additional predicted binding sites for cellular miRNAs, including 2 predicted sites for miR-150 on the minus-strand transcript coding for HBZ (80); the potential for miR-150 to regulate HBZ expression remains to be explored.

### Mechanisms involved in repression of miR-150 expression/function

Mechanisms known to repress miR-150 expression/function include promoter methylation, interference with miRNA precursor processing, inhibition by lncRNAs (long noncoding RNAs), and release from the cell.

The miR-150 stem-loop is coded on the minus strand of chromosome 19q13.33 (chr19: 49,500,785-49,500,868 [-]). An investigation of a ~ 500-bp sequence spanning this region identified several CpG dinucleotides that are targets for methylation by DNMT1 and mediate miR-150 silencing in anaplastic large-cell lymphoma cells (81).

Early studies of miRNA regulation showed that MYC represses expression of many miRNAs, including miR-150 (82). Further investigations revealed a control circuit in which MYC induces expression of LIN28, an RNA-binding protein that interferes with miRNA maturation, including that of miR-150. This leads to deregulation of oncogenes normally targeted by miR-150, including MYB and FLT3 (83, 84). The impact of MYC on miR-150 may be cell-context dependent, as MYC was shown to repress miR-150 expression independently of LIN28 in follicular lymphoma cells (85). The role of MYC in miR-150 transcription/processing may be relevant in HTLV-1 infection, given the findings that MYC expression and function are increased through the activities of the viral regulatory proteins HBZ and p30, respectively (86, 87).

The multiple functions of lncRNAs include direct miRNA binding, which ‘sponges’ the miRNA from its 3’UTR targets. An investigation of lncRNAs in a panel of HTLV-1-transformed- and ATLL cell lines revealed expression of AVRIL, HOTAIR H19, TUSC7, MALAT1 and SAF lncRNAs and demonstrated roles for AVRIL in NF-κB signaling and repression of CDKN1A expression (88). MALAT1 is also a good candidate for further study, as it was recently shown to act as a sponge for miR-150 in non-Hodgkin lymphoma (89).

Many miRNAs, including miR-150, are released into the extracellular environment as cargo in extracellular vesicles (EVs) or in association with Argonaute proteins or high-density lipoproteins (HDL) [reviewed by (90)]. Extracellular release of miRNAs can serve as a mechanism to rapidly eliminate them from the cell, or to target them to recipient cells.

Studies of CD4+ T-cells indicated that the rapid decline in miR-150 levels induced by *in vitro* stimulation is associated with its active export in EVs and upregulation of MYB expression (91). Tregs also actively export miR-150 in EVs which can in turn induce downregulation of MYB in recipient cells and reduce the proliferation of *in vitro*-stimulated CD4+ T-cells (92).

HTLV-1-infected cell lines are known to release EVs that contain Tax protein, a subset of viral mRNAs, and cell signaling molecules [reviewed by (93)]. These EVs can have activating effects on recipient cells and promote cell-cell contact, thereby facilitating virus transmission (94). It would be interesting to determine whether HTLV-1-infected cells release miR-150 as a means of depleting their intracellular pools or to provide signals to surrounding cells.

## Conclusions and perspectives

Future studies aimed at defining the expression pattern of miR-150 and its targets in HTLV-1-infected- and ATLL cells will help complete the picture of the interplay between the miRNA regulatory network and HTLV-1 infection and pathogenesis. An important task will be to extend miR-150 expression analyses to primary ATLL samples and PBMC from asymptomatic patients to specific populations enriched for CD4+, infected cells.

**Figure 2:**
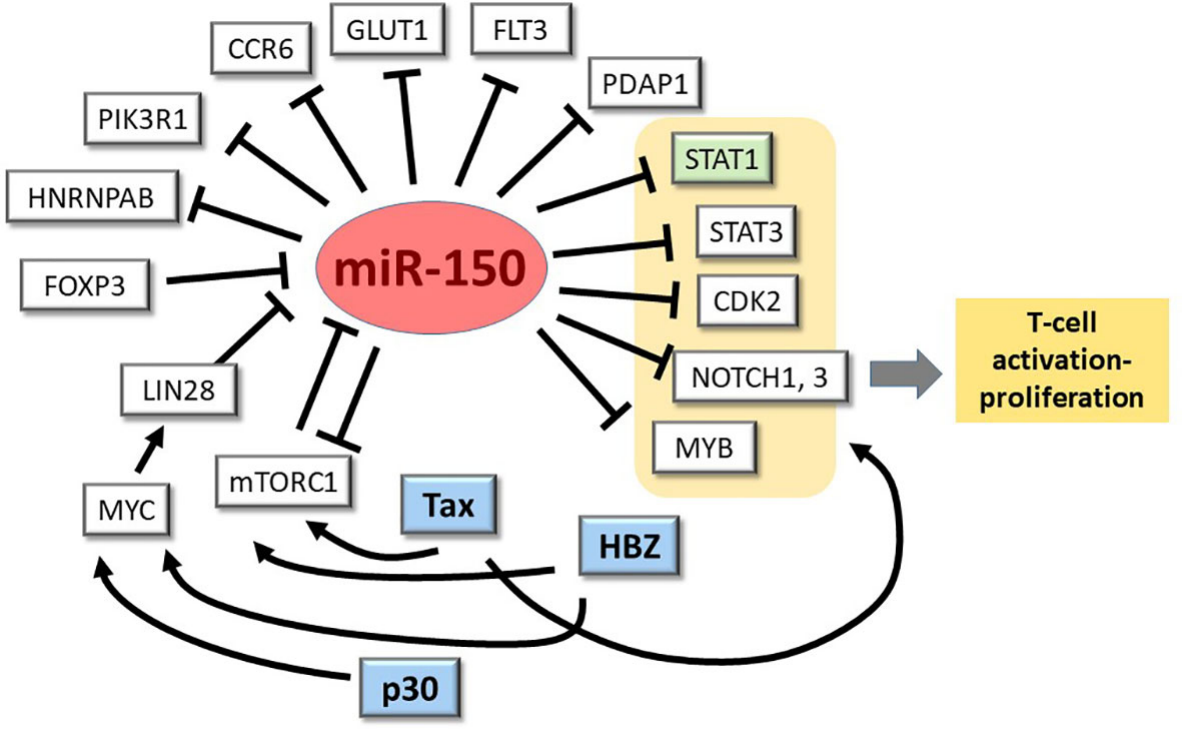
miR-150-mRNA interactions in HTLV-1 infection/transformation: one confirmed, many to explore. miR-150 is known to repress expression of STAT1 (highlighted in green) when overexpressed in ATLL cell lines (24). As depicted here and described in the text, many other genes of potential relevance to HTLV-1 infection and pathogenesis are known to be controlled by miR-150 in normal and neoplastic T-cells and other leukocyte populations.

The strong downregulation of miR-150 observed in many HTLV-1-transformed- and ATLL-derived cell lines makes these lines useful models for discovering miR-150 targets that are relevant to T-cells and the virus, STAT1 being the first such example (26) (see **Figure 2**). An *in vitro*-co-cultivation system could be employed to verify some of the mRNA targets described above, e.g. GLUT1, and to test the effects of forced expression/silencing of miR-150 on HTLV-1 infection and immortalization. The results of these studies will be instrumental to assess the potential relevance of miR-150 as a biomarker and possible therapeutic target in HTLV-1 infection and disease.

## Author contributions

DD’A, VR, MS-B, and VC wrote the manuscript. MS-B and DD’A prepared **Figure 1**. VC and DD’A prepared **Figure 2**. All authors contributed to the article and approved the submitted version.

## Funding

Investigator Grant #24935, from AIRC (VC) and BIRD #204551/20 from the Department of Biomedical Sciences, University of Padova (DD’A).

## Conflict of interest

The authors declare that the research was conducted in the absence of any commercial or financial relationships that could be construed as a potential conflict of interest.

## Publisher’s note

All claims expressed in this article are solely those of the authors and do not necessarily represent those of their affiliated organizations, or those of the publisher, the editors and the reviewers. Any product that may be evaluated in this article, or claim that may be made by its manufacturer, is not guaranteed or endorsed by the publisher.
